# Comparative Evaluation of Colistin Broth Disk Elution Method With Two Commercially Available Systems for Colistin Susceptibility Testing Against Carbapenem-Resistant Klebsiella pneumoniae: A Single-Center Exploratory Study

**DOI:** 10.7759/cureus.25549

**Published:** 2022-05-31

**Authors:** Arpana Singh, Mohit Bhatia, Sasi Rekha, Diksha Rani, Pratiksha Kamboj, Deepika Chakraborty, Pratima Gupta

**Affiliations:** 1 Microbiology, All India Institute of Medical Sciences Rishikesh, Rishikesh, IND

**Keywords:** carbapenem-resistant, broth micro dilution, klebsiella pneumoniae, india, colistin broth disk elution

## Abstract

Purpose: The purpose is to explore the diagnostic utility of colistin broth disk elution (CBDE) as a simple and reliable method of colistin susceptibility testing.

Materials and methods: An exploratory study was undertaken in a tertiary care teaching hospital in Uttarakhand, from September 2021 to March 2022, after obtaining approval from the Institute Ethics Committee. Twenty-five non-repetitive carbapenem-resistant *Klebsiella pneumoniae *clinical isolates were included in the study. Matrix‐assisted laser desorption ionization-time-of-flight mass spectrometry (MALDI-TOF MS) and BD Phoenix M50 system were used to perform species-level identification and antibiotic susceptibility testing (AST), respectively, as per the manufacturer’s instructions. AST results (including those of colistin) were interpreted as per the CLSI guidelines 2022. The test isolates were further subjected to additional in vitro colistin susceptibility testing using a commercially available Mikrolatest colistin susceptibility testing kit and CBDE, respectively.

Results: The in vitro colistin resistance rates varied from 8% by BD Phoenix system to 20% by Mikrolatest kit and 32% by CBDE, respectively. For colistin susceptibility, a higher CA was observed between the BD Phoenix system and CBDE (64.71%) than between the Mikrolatest kit and CBDE (31.60%). Overall, a statistically significant fair agreement was observed between the BD Phoenix system and CBDE (Kappa: 0.312; 95% CI: 0.036 to 0.660) and Mikrolatest MIC colistin kit and CBDE (Kappa: 0.286; 95% CI: 0.111 to 0.683), respectively.

Conclusions: In vitro colistin testing remains a significant challenge globally. Although the present study results are inconclusive due to the small sample size, we should conduct multi-centric studies globally, taking a considerable sample size representing different Gram-negative bacilli to generate conclusive evidence on the utility of CBDE as a reliable method of colistin susceptibility testing.

## Introduction

In recent years, there has been a surge in reports of infections due to carbapenem-resistant Enterobacteriaceae (CRE) strains [1]. Dealing with CRE strains is challenging for the following reasons: (a) Owing to the multi-drug resistant (MDR) nature of these isolates, treatment options for CRE-induced infections are limited. (b) These microorganisms have the potential to cause nosocomial outbreaks. (c) CRE infections are associated with a high prevalence of morbidity and mortality. Carbapenem-resistant *Klebsiella pneumoniae* (CRKP) are species of CRE that have been listed as one of the critical priority antibiotic-resistant bacterial pathogens by the World Health Organization (WHO) [1,2]. First reported in the 1990s, CRKP has become a significant threat to hospitalized patients due to high mortality rates, prolonged duration of hospitalization, and increased treatment costs [3,4]. *K. pneumoniae* has been found as a significant entry site for antibiotic resistance genes into the Enterobacterales species [2].

Some Indian studies have revealed a varied prevalence of carbapenem resistance (35 to 75%) in *K. pneumoniae* [5-7]. Due to the resistance of this pathogen to different antibiotic classes, there is a renewed interest in polymyxins (including colistin) as the best available treatment option in the Indian sub-continent. Despite drawbacks like a limited antibacterial spectrum and nephrotoxicity, this age-old antibiotic is increasingly being used to treat life-threatening infections caused by multidrug-resistant Gram-negative bacteria like CRKP [8,9]. Colistin resistance has been reported in Gram-negative bacteria all over the world [8]. While careless use of this drug as a last resort antibiotic may partially explain this phenomenon, the use of non-standardized in vitro colistin susceptibility testing and reporting methods by medical laboratories could have majorly contributed to this “untoward surge” [8].

Clinical laboratories have struggled to perform colistin susceptibility testing in the past. According to the Clinical Laboratory Standards Institute (CLSI) and the European Committee on Antimicrobial Susceptibility Testing (EUCAST), the only validated test method for polymyxins is reference broth microdilution (rBMD), which is time-consuming and performed in only a few clinical laboratories [10]. Various colistin susceptibility testing methods, like automated bacterial ID/AST systems, E-test, agar dilution, and disk diffusion, have been used over the years. Studies based on the diagnostic evaluation of these methods, taking rBMD as the gold standard, have also been carried out in different parts of the world. Variable results have so far been obtained from these studies, further emphasizing the need for developing rapid, user friendly, and accurate in vitro colistin susceptibility testing methods [11,12].

The CLSI formed an ad hoc working group (ahWG) to solve the polymyxin testing difficulties. This group examined the utility of two MIC-based alternative methods for testing colistin namely, colistin broth disk elution (CBDE) and colistin agar test (CAT). The goal of evaluating these tests was to create a procedure that was as precise as rBMD but was more suitable for routine clinical laboratory usage, i.e., one that could be performed using commonly available materials and was easy to perform [10]. The CBDE employs commercially available colistin disks, which are introduced to commercially supplied pre-aliquoted cation-adjusted Mueller-Hinton broth (CA-MHB) to achieve a predetermined colistin concentration. Although diagnostic evaluation of this method has been performed to some extent in different countries [10,13,14], there is a lack of similar data from the Indian subcontinent. This single-center study was performed to explore the utility of CBDE as a simple and reliable method of colistin susceptibility testing in CR-KP clinical isolates.

## Materials and methods

An exploratory study was carried out in a tertiary care teaching hospital in Uttarakhand from September 2021 to March 2022, after receiving approval from the Institute Ethics Committee of All India Institute of Medical Sciences (Registration No. ECR/736/Inst/UK/2015/RR-18) vide approval letter No-AIIMS/IEC/21/512.

We only included CRKP clinical isolates in the study. These isolates were also MDR. All other Gram-negative and Gram-positive bacterial isolates were excluded from the study. The main goal of this study was to compare the in vitro colistin susceptibility of CR-KP using the BD Phoenix M50 automated ID/AST system, Mikrolatest colistin susceptibility testing kit, and the CBDE method.

Twenty-five non-repetitive CRKP isolated from pus (n=9), urine (n=8), and blood (n=8) samples of inpatients were collected from February to March 2021 and stocked for testing later. Matrix‐assisted laser desorption ionization-time-of-flight mass spectrometry (MALDI-TOF MS) (Bruker Daltonik GmbH, Germany) and NMIC/ID 55 panel of BD Phoenix M50 ID/AST system (Becton Dickinson, Maryland, USA) were used to perform species-level identification and antibiotic susceptibility testing (AST), respectively, as per manufacturer’s instructions. AST results (including those of colistin) were interpreted as per the Clinical and Laboratory Standards Institute (CLSI) guidelines 2022 [15]. The test isolates were further subjected to additional in vitro colistin susceptibility testing using a commercially available Mikrolatest colistin susceptibility testing kit (Transasia Bio-Medicals, Mumbai, India) and CBDE, respectively. Each of these additional tests was performed by the same technical staff only once per isolate.

**Figure 1:**
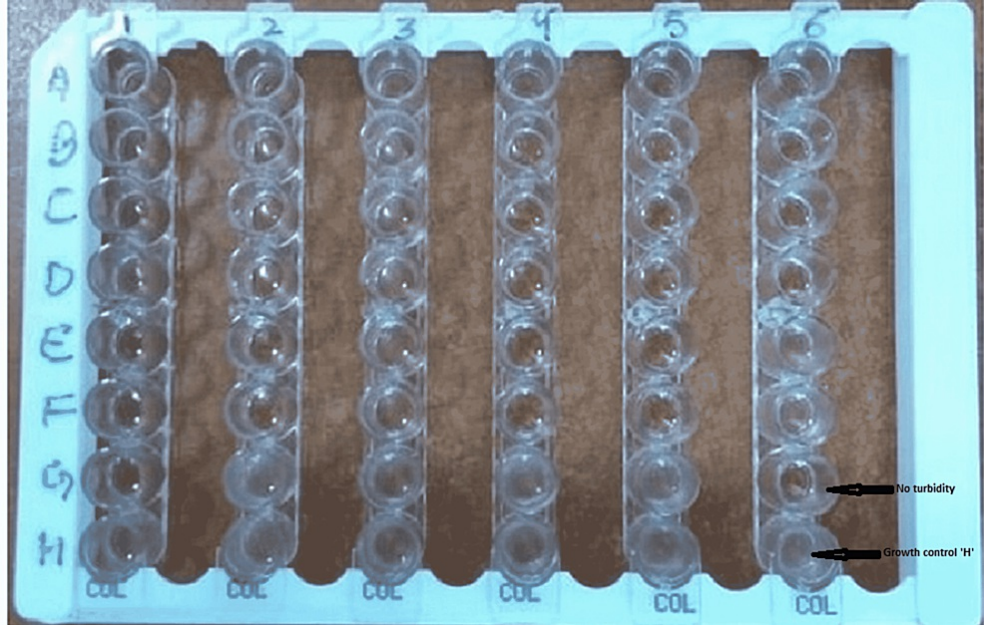
Mikrolatest kit based broth micro dilution test Colistin concentration doubled from wells “G” to “A,” respectively (0.25-16 mg/L), with “H” well serving as growth control. The first well position from bottom to top, showing no growth in any form (any granulation, button, or turbidity) was considered MIC value.

The Mikrolatest kit is based on the broth microdilution principle. It is designed to test the susceptibility of Gram-negative bacteria to colistin based on MIC determination. This test encompasses the rehydration of antibiotics in the wells with CA-MHB and bacterial suspension, as depicted in Figure 1. The CLSI-approved CBDE method was considered the reference test for this study. Four screw-capped borosilicate tubes containing 10 mL CA-MHB (Microxpress, Tulip Diagnostics Pvt. Ltd., Goa, India) were labeled as 1, 2, 4 µg/mL, and control, respectively, for this test. Ten µg colistin sulfate disks (Microxpress) kept at room temperature, were added to the first three tubes using an aseptic technique to obtain final concentrations of 1 µg/mL (one disk in 10 mL), 2 µg/mL (two disks in 10mL) and four µg/mL (four disks in 10 mL), respectively. No disks were added to the control tube (0 µg/mL; 0 disks in 10 mL).

**Figure 2:**
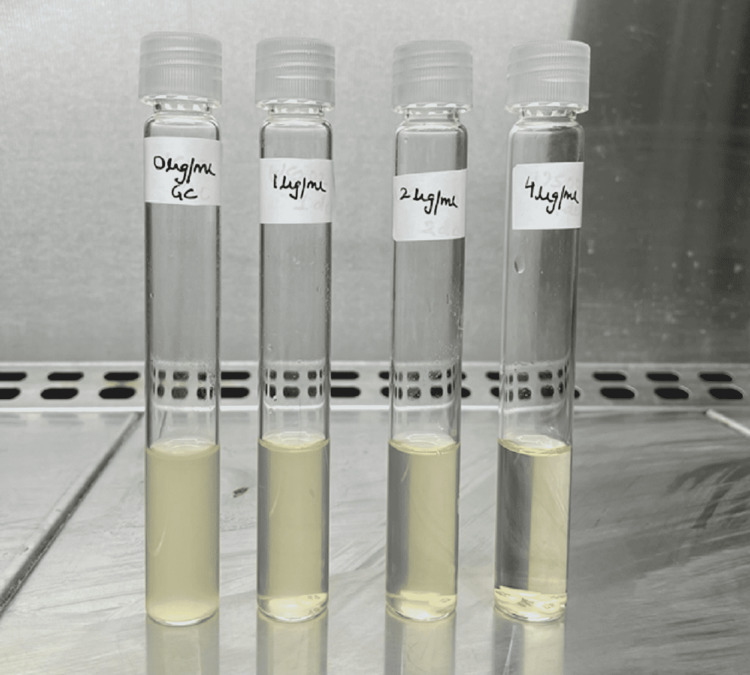
Colistin broth disk elution method

Colistin was allowed to elute from the antibiotic disks for at least 30 minutes and no longer than 60 minutes at room temperature after the tubes holding the antibiotic disks were gently vortexed. A standardized inoculum was prepared for each test isolate by picking three to five colonies with a sterile loop from fresh (18-24 hours) non-selective agar plate and transferring the same to 5 mL sterile saline, turbidity of which was equivalent of a 0.5 McFarland standard. 50 µL of standardized inoculum was added to all four tubes to attain a final inoculums concentration of approximately 7.5 x 10^5^ CFU/mL per tube. As a purity check, subculture from the original inoculum tube to a blood agar plate was performed using a 10 µL loop. Each inoculated tube was tightly capped and vortexed at slow speed for adequately mixing the contents, ensuring that the colistin disks did not adhere to the cap or the glass surface above the liquid meniscus. After vortexing, all tubes (with slightly loosened caps) along with the purity plate were incubated at 33-35°C in ambient air for 16-20 hours. The results were evaluated after analyzing the purity plate to determine that the inoculum was pure after incubation. For the test to be valid, the presence of noticeable turbidity in the growth control tube was a prerequisite. The lowest concentration that totally inhibited the growth of the test isolate was interpreted as the minimum inhibitory concentration (MIC) as shown in Figure 2.

*Escherichia coli* ATCC 25922, *Pseudomonas aeruginosa* ATCC 27853 and *mcr*-1 positive *E. coli* NCTC 13846 (Kwik Stik, Microbiologics, USA) were used as control strains for all three aforementioned testing methods. The Bruker bacterial test standard (BTS) was used for performing quality checks in MALDI-TOF MS.

Discrepancies in colistin susceptibility results were categorized as very major errors (VMEs) and major errors (MEs), for calculating the categorical agreement (CA). VMEs were defined as the bacterial isolates labelled as susceptible (S) by the tests under evaluation (BD Phoenix M50 ID/AST system and/or Mikrolatest kit), and resistant (R) by the reference test (CBDE) [16]. Major errors were defined as the bacterial isolates labelled as resistant by BD Phoenix M50 ID/AST system and/or Mikrolatest kit, and susceptible by the reference test [16]. Please note that the categories used for reporting colistin susceptibility test results as per CLSI 2022 guidelines are intermediate (I) and resistant (R) only, in contrast to the EUCAST 2022 guidelines, in which there is a susceptible category (S) in place of “I” [15,17]. For calculating ME and VME results of colistin susceptibility testing in the present study, “I” of CLSI was considered equivalent to “S” of EUCAST guidelines. The coefficient of agreement (Kappa) between the three test methods w.r.t. colistin susceptibility test results was calculated using Graphpad Prism 9.1.2 (226) software (San Diego, CA, USA).

## Results

All test isolates were resistant to ciprofloxacin, levofloxacin, ampicillin, piperacillin, piperacillin-tazobactam, amoxicillin-clavulanate, aztreonam, cefuroxime, ceftriaxone, cefepime, ceftazidime, cefazolin, imipenem, meropenem and co-trimoxazole. Very high in vitro resistance (96%; 24/25) to amikacin and gentamicin was observed. 72% (18/25) and 32% (8/25) CRKP strains exhibited in vitro resistance to tetracycline and chloramphenicol, respectively.

Colistin susceptibility test results of test isolates and control strains using the BD Phoenix system, Mikrolatest kit, and CBDE, respectively, have been depicted in Table 1. The test isolates were classified as colistin intermediate (MIC ≤ 2 µg/mL) or resistant (MIC ≥ 4 µg/mL), respectively, as per CLSI 2022 guidelines [15].

**Table 1:** Colistin susceptibility test results of all bacterial isolates as determined by BD Phoenix M50 system, Mikrolatest kit and colistin broth disk elution method *I/R: Intermediate/Resistant; MIC: Minimum inhibitory concentration

Organisms	BD Phoenix M50 ID/AST system		Mikrolatest MIC colistin susceptibility testing kit		Colistin broth disk elution test	
	Colistin MIC (µg/mL)	I/R*	Colistin MIC (µg/mL)	I/R*	Colistin MIC (µg/mL)	I/R*
CONTROL STRAINS						
ATCC 27853 Pseudomonas aeruginosa	<2	I	<2	I	≤1	I
ATCC 25922 *E. coli*	<2	I	<2	I	≤1	I
NCTC 13846 *E. coli* (mcr-1 positive)	4	R	4	R	≤4	R
TEST ISOLATES OBTAINED FROM SAMPLE TYPES						
CRKP 1(Urine)	≤0.5	I	≤0.5	I	≤2	I
CRKP 2(Pus)	≤0.5	I	≤0.5	I	≤1	I
CRKP 3(Pus)	≤0.5	I	≤0.5	I	≤2	I
CRKP 4(Pus)	≤0.5	I	>16	R	≥4	R
CRKP 5(Blood)	≤0.5	I	≤0.25	I	≤1	I
CRKP 6(Blood)	≤0.5	I	≤0.25	I	≤1	I
CRKP 7(Blood)	≤0.5	I	≤0.5	I	≤1	I
CRKP 8(Blood)	≤0.5	I	≤0.25	I	≤2	I
CR-Kp 9(Pus)	≤0.5	I	≤0.25	I	≤1	I
CRKP 10(Pus)	≤0.5	I	≤0.5	I	≤1	I
CRKP11(Blood)	≤0.5	I	≤0.25	I	≤1	I
CRKP 12(Urine)	≤0.5	I	≤1	I	≤1	I
CRKP 13(Pus)	≤0.5	I	≤0.5	I	≤1	I
CRKP 14(Pus)	≤0.5	I	>16	R	≤2	I
CRKP 15(Urine)	≤0.5	I	≤0.5	I	≤1	I
CRKP 16(Pus)	≤0.5	I	≤0.5	I	≥4	R
CRKP 17(Blood)	≤0.5	I	≤0.5	I	≤1	I
CRKP 18(Urine)	≤0.5	I	≤0.5	I	≥4	R
CRKP 19(Urine)	≤0.5	I	≤0.25	I	≥4	R
CRKP 20(Urine)	≥4	R	>16	R	≥4	R
CRKP 21(Blood)	≤0.5	I	≤0.5	I	≤2	I
CRKP 22(Urine)	≥4	R	>16	R	≥4	R
CRKP 23(Pus)	≤0.5	I	≤0.25	I	≤1	I
CRKP 24(Blood)	≤0.5	I	≤0.25	I	≥4	R
CRKP 25(Urine)	≤0.5	I	≤0.5	I	≥4	R

Colistin MIC of ATCC 25922 *E. coli *(reference range: 0.25-2µg/mL) and ATCC 27853 *P. aeruginosa* (reference range: 0.5-5 µg/mL) were recorded as per CLSI guidelines 2022 [15]. Colistin MIC of *mcr*-1 positive NCTC 13846 *E. coli *(4 µg/mL; reference range: 2-8 µg/mL) was evaluated as per European Committee on Antimicrobial Susceptibility Testing (EUCAST) guidelines 2022 [17]. These three served as QC strains for in vitro testing by the BD Phoenix system and Mikrolatest kit, respectively. As per CLSI 2022 guidelines, only two QC strains, namely ATCC 27853 *P. aeruginosa* (MIC reference range: ≤1-4 µg/mL) and AR Bank #0349*mcr-*1 strain (MIC reference range: ≤1-4 µg/mL), have been recommended while performing CBDE [15]. Owing to the non-availability of AR Bank #0349*mcr*-1 strain, the mcr-1 positive NCTC 13846 *E. coli *strain was used instead. ATCC 25922 *E. coli* was also tested by CBDE in addition to the recommended QC strains.

Table 2 shows the colistin resistance pattern (expressed as a percentage) of all test isolates as determined by different methods, along with the agreement analysis (categorical agreement and Cohen’s Kappa). For colistin susceptibility, a higher CA was observed between BD Phoenix system and CBDE (64.71%) than between Mikrolatest kit and CBDE (31.60%). Overall, a statistically significant fair agreement was observed between BD Phoenix system and CBDE (Kappa: 0.312; 95% CI: 0.036 to 0.660) and Mikrolatest MIC colistin kit and CBDE (Kappa: 0.286; 95% CI: 0.111 to 0.683), respectively.

**Table 2:** Summary of colistin resistance rates of the test isolates and agreement analysis (Categorical agreement and Cohen's Kappa) of the three colistin susceptibility testing methods used in the study n: Number of resistant isolates, N: Total number of isolates, ME: Major error (Colistin resistant by T1 and/or T2 and susceptible by reference test [T3]; [No. of major discrepancies/Total no. of susceptible organisms by reference method]*100), VME: Very major error (Colistin susceptible by T1 and/or T2 and resistant by reference test [T3] expressed as %; [No. of very major discrepancies/Total no. of resistant organisms by reference method]*100)

Test Organism	BD Phoenix M50 ID/AST system (T1) n/N (%)	Mikrolatest MIC colistin susceptibility testing kit (T2) n/N (%)	Colistin Broth disk elution Test (T3) n/N (%) (Reference method)	Errors				Categorical agreement (%)		Kappa value	
				ME (%)		VME (%)		T1 vs T3	T2 vs T3	T1 vs T3	T2 vs T3
				T1 vs T3	T2 vs T3	T1 vs T3	T2 vs T3				
CR-Kp	2/25(8)	4/25(16)	8/25(32)	0	5.9	35.29	62.5	64.71	31.60	0.312	0.286

## Discussion

India has a high burden of infectious illnesses and is one of the world's top antibiotic consumers [18]. The emergence of resistance to the newer and more expensive drugs like carbapenems, in addition to the older and more frequently used classes of antibiotics, is worrisome. Carbapenems are widely considered to be last-resort antibiotics and are used when first and second-line treatment options have failed [19]. As per the National antimicrobial resistance surveillance network (NARS-Net India) AMR annual report 2021, high resistance levels to carbapenem antibiotics (imipenem: 41.05%, ertapenem: 45.64%, and meropenem: 41.44%) have been observed among *Klebsiella* spp. In addition to this, alarmingly high rates of resistance to other antibiotics like amikacin (44.8%), piperacillin-tazobactam (57.9%), co-trimoxazole (63.1%), cefepime (69.1%), ciprofloxacin (62.1%), and cefotaxime (79.5%), have also been documented in *Klebsiella* spp. [20]. In the present study, extremely high rates of antibiotic resistance were observed in all test isolates. These findings reflect the current paradigm of antibiotic resistance in the Indian subcontinent. 

The emergence of colistin resistance poses a significant threat in countries with high rates of infections due to CRE. Due to the scarcity of data on colistin resistance in India, it is critical to conduct hospital-based surveillance studies on colistin resistance among Gram-negative bacteria. According to the NARS-Net India AMR annual report 2021, colistin resistance (determined by rBMD method) among *E. coli*, *Klebsiella* spp., *Pseudomonas* spp., and *Acinetobacter* spp. has been documented as 3.1%, 7.2%, 5.8%, and 4.7%, respectively [20]. In the present study, the in vitro colistin resistance rates varied from 8% by the BD Phoenix system to 20% by the Mikrolatest kit and 32% by CBDE, respectively. This disparity could be due to differences in testing methodologies or the heteroresistance phenomenon [21].

The BD Phoenix M50 ID/AST system and CBDE had a 64.71% categorical agreement in our study. In a study done by Shams et al., a higher CA (95.24%) was observed between the BD Phoenix system and the rBMD method when compared to that between CBDE and the rBMD method (88.89%) [22].In a study conducted by Ozyurt et al., it was observed that the BD Phoenix system did not reliably distinguish colistin-resistant and colistin-susceptible strains (CA: 95% w.r.t. rBMD) [13]. A noteworthy point is that the CA between the BD Phoenix system and CBDE was not calculated in the studies mentioned above. 

The present study's CA between the Mikrolatest MIC colistin kit and CBDE was 31.60%. To the best of our knowledge, similar studies comparing these two testing methodologies are not available. In a comparative evaluation study conducted by Singh et al., the CA between the Mikrolatest kit and the MicroScan Walkaway 96 Plus ID/AST system w.r.t. colistin susceptibility test results was 71.4%, 85.7%, and 100% for *Acinetobacter baumannii*, *P. aeruginosa*, and *K. pneumoniae*, respectively [21]. Sarumathi et al. compared the Mikrolatest kit with the in-house reference broth microdilution method. In this study, CA between these two methods was 91.1%, with VMEs and MEs being 2.7% and 6.1%, respectively [23].

Based on the results of a multi-centric study by Simner et al., CBDE was provisionally approved for testing of Enterobacterales and *P. aeruginosa* by the CLSI antimicrobial susceptibility testing (AST) Subcommittee in 2019. Overall, 94.4% and 97.9% of CBDE results were in essential and categorical agreement, respectively, with rBMD MICs [10]. Some of the concerns about using this method, as noted by Simner et al., are that CBDE is a low-throughput test that requires numerous disks and tubes for testing each bacterial isolate along with a sufficiently large space for incubator. The CLSI recommended QC strains for colistin (*Escherichia coli* ATCC 25922 and *P. aeruginosa* ATCC 28753) may yield off-scale MICs with CBDE. Colistin and polymyxin B have presented QC challenges at CLSI meetings in the past. Because of the CBDE's complicated QC background, the ahWG foresaw QC issues and attempted to establish a new QC strain, *Escherichia coli* AR Bank no. 0349. MICs ranging from 2 to 4 µg/mL have been consistently obtained with this strain by various methods. Only one brand of CA-MHB and colistin disk was used for evaluating CBDE. As per the CLSI standard M23, evaluation of different brands of media/disks for new test methods is not recommended. However, given the issues connected with colistin testing that have been recorded to date, this concern is noteworthy. CBDE cannot be used to test polymyxin B. However, CLSI has recently confirmed that colistin results could be used to predict those of polymyxin B [10].

The present study had several limitations, like small sample size, non-structured sampling technique, and non-inclusion of *Pseudomonas* spp., *Acinetobacter* spp., and Enterobacterales spp. other than *K. pneumoniae*. Several technical issues also could have led to discrepant results, like *Escherichia coli* NCTC 13846 was used as QC strain instead of the recommended *Escherichia coli* AR Bank no. 0349 for CBDE. The bacterial isolates, including QC strains, were not tested in duplicate or triplicate. None of these tests was performed by more than one person; therefore, we could not ascertain the repeatability and reproducibility of the test results. We did not use the rBMD method for determining colistin susceptibility in our study. The noteworthy point is that the CBDE method depends on the quality of colistin disks and cation adjusted Mueller Hinton broth. We restricted our analysis to using the reagents of one brand. However, one can hypothesize that the susceptibility test results of the CBDE test may vary when colistin disks and MH broth of different brands are used. This aspect was also not looked into in our study.

## Conclusions

To conclude, we would like to reiterate that in vitro colistin testing remains a significant challenge worldwide. The technical limitations of our study are another potential area of research we need to dwell on. Although the present study results are inconclusive due to the small sample size, we should conduct multi-centric studies globally, taking a considerable sample size representing different Gram-negative bacilli to generate conclusive evidence on the utility of CBDE as a reliable method of colistin susceptibility testing. We are hopeful that both CLSI and EUCAST will be able to provide some updates on CBDE in the years to come, which will further advance our understanding of the diagnostic usefulness of the CBD method.
